# Case Report: Temporary supratrochanteric plating for the management of subtrochanteric femoral fracture in an immature dog

**DOI:** 10.3389/fvets.2025.1703404

**Published:** 2025-12-03

**Authors:** Hojung Bae, Jongpil Yoon, Youngjin Jeon, Haebeom Lee, Jaemin Jeong

**Affiliations:** College of Veterinary Medicine, Chungnam National University, Daejeon, Republic of Korea

**Keywords:** temporary supratrochanteric plating, subtrochanteric femoral fracture, immature dog, trochanteric apophysis, greater trochanter

## Abstract

This case report describes the successful management of a subtrochanteric femoral fracture in a 2-month-old dog using temporary supratrochanteric plate fixation, performed prior to the development of the greater trochanter apophysis. A tibial plateau leveling osteotomy plate was applied to the proximolateral femur and subsequently removed following radiographic evidence of bridging callus formation. At the 21-month follow-up, the dog exhibited normal limb function, symmetrical femoral length, and appropriate development of the greater trochanter. Although a mild procurvatum deformity was noted, no clinical signs or range of motion deficits were observed. These findings support the use of temporary fixation across the trochanteric region as a feasible treatment strategy for immature subtrochanteric femoral fractures when performed prior to apophyseal development.

## 1 Introduction

Subtrochanteric femoral fractures represent a relatively uncommon type of femoral injury involving the proximal metaphysis distal to the trochanters (1). Conservative management is generally associated with suboptimal functional outcomes; therefore, surgical stabilization is typically indicated. Surgical management options for subtrochanteric femoral fracture include bone plating, interlocking nailing, and external skeletal fixation, with the choice of technique determined by factors such as the patient's age, the interval between trauma and intervention (2, 3), and the anatomical characteristics of the fracture (4).

Surgical management of subtrochanteric fractures presents several technical challenges. The limited bone stock available in the short proximal segment often precludes secure screw or locking bolt purchase, thereby complicating stable fixation (3). In addition, strong deforming forces generated by muscles inserting on the proximal femur, such as the gluteal muscles, obturators, and iliopsoas, can interfere with fracture reduction and increase the risk of fixation failure. Especially in immature patients, additional challenges arise due to the need to preserve the residual growth potential of the capital physis and the trochanteric apophysis (5).

Trochanteric physis is an apophysis that develops at approximately 3 months of age in dogs, in response to tensile forces generated by tendon attachment (6). Although its contribution to the longitudinal femoral growth is minimal (7), it is associated with shaping of the proximal femur, and damage to it may alter hip joint biomechanics and predispose to degenerative joint disease of the hip (4, 8). Prior to the formation of the secondary ossification center, the trochanteric region is composed entirely of cartilaginous tissue (9). To the authors' knowledge, no veterinary studies have described fracture repair techniques involving implant placement across this region before apophyseal formation.

This case report describes the surgical management of a subtrochanteric femoral fracture in an immature dog using temporary supratrochanteric plate fixation performed prior to the formation of the trochanteric apophysis. This approach aimed to achieve rigid fixation while minimizing interference with the developing apophyseal region, thereby preserving growth potential in the proximal femur. The long-term outcome, including femoral growth, morphological alteration of the femur, limb function, and greater trochanter development, was evaluated over an extended follow-up period.

## 2 Case description

A 2-month-old, female mongrel dog was presented to the veterinary teaching hospital of Chungnam National University with a of suspected history of trauma sustained 2–3 days prior to admission. Upon presentation, the patient exhibited non-weight-bearing lameness of the left pelvic limb. Pain was elicited on palpation of the left femoral region.

**Figure 1:**
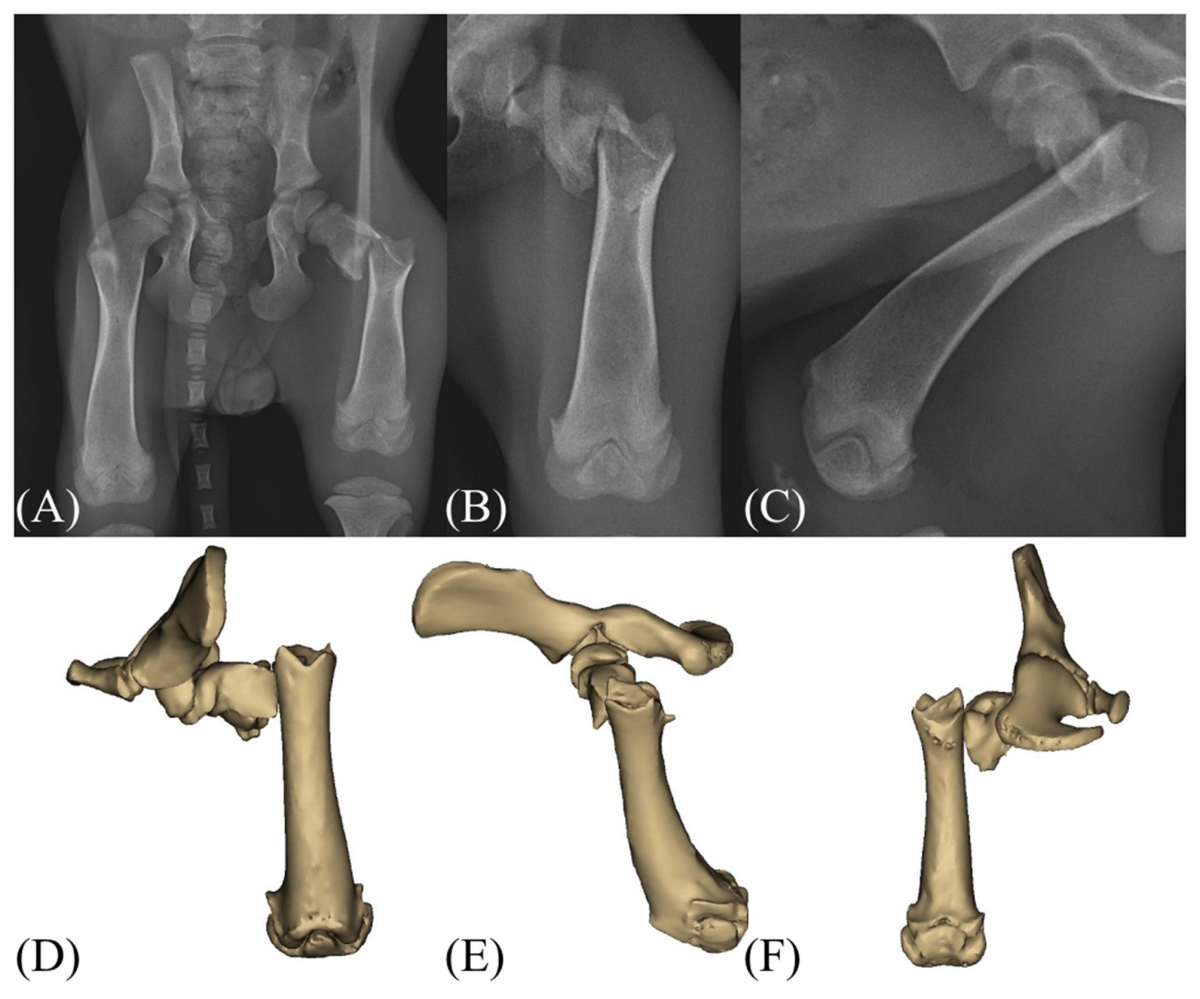
Preoperative radiograph and CT images of the patient. **(A–C)** Radiographs of the left pelvic limb demonstrating a transverse subtrochanteric fracture of the femur with proximolateral displacement of the distal fragment. **(D–F)** Computed tomography images confirming the subtrochanteric fracture configuration and assisting in preoperative planning.

Radiographic examination revealed a closed, simple, transverse subtrochanteric fracture of the left femur with the distal fragment displaced proximolaterally (Figure 1). Additional findings included fracture of the left 6th to 10th ribs and a left calvarial fracture with minimal displacement. Abdominal ultrasonography demonstrated evidence of blunt trauma to the left kidney, hematoperitoneum, and the presence of an inguinal hernia. Computed tomography was performed for further characterization of the femoral fracture (Figure 1).

Despite the presence of multiple injuries, the absence of neurovascular compromise and the patient's young age were considered favorable prognostic indicators for functional recovery. Surgical repair of the subtrochanteric fracture was performed 3 days after presentation, following clinical stabilization of the patient's systemic condition, including improvement of renal trauma, hematoperitoneum.

For anesthesia, midazolam (0.2 mg/kg IV) was used for premedication, and propofol (4 mg/kg IV) isoflurane were used for induction and maintenance, respectively. Remifentanil (6–18 μg/kg/h IV) was administered for intraoperative analgesia, and cefazolin (22 mg/kg IV) was administered 30 min prior to the skin incision and subsequently every 90 min during the procedure as a prophylactic antibiotic.

**Figure 2:**
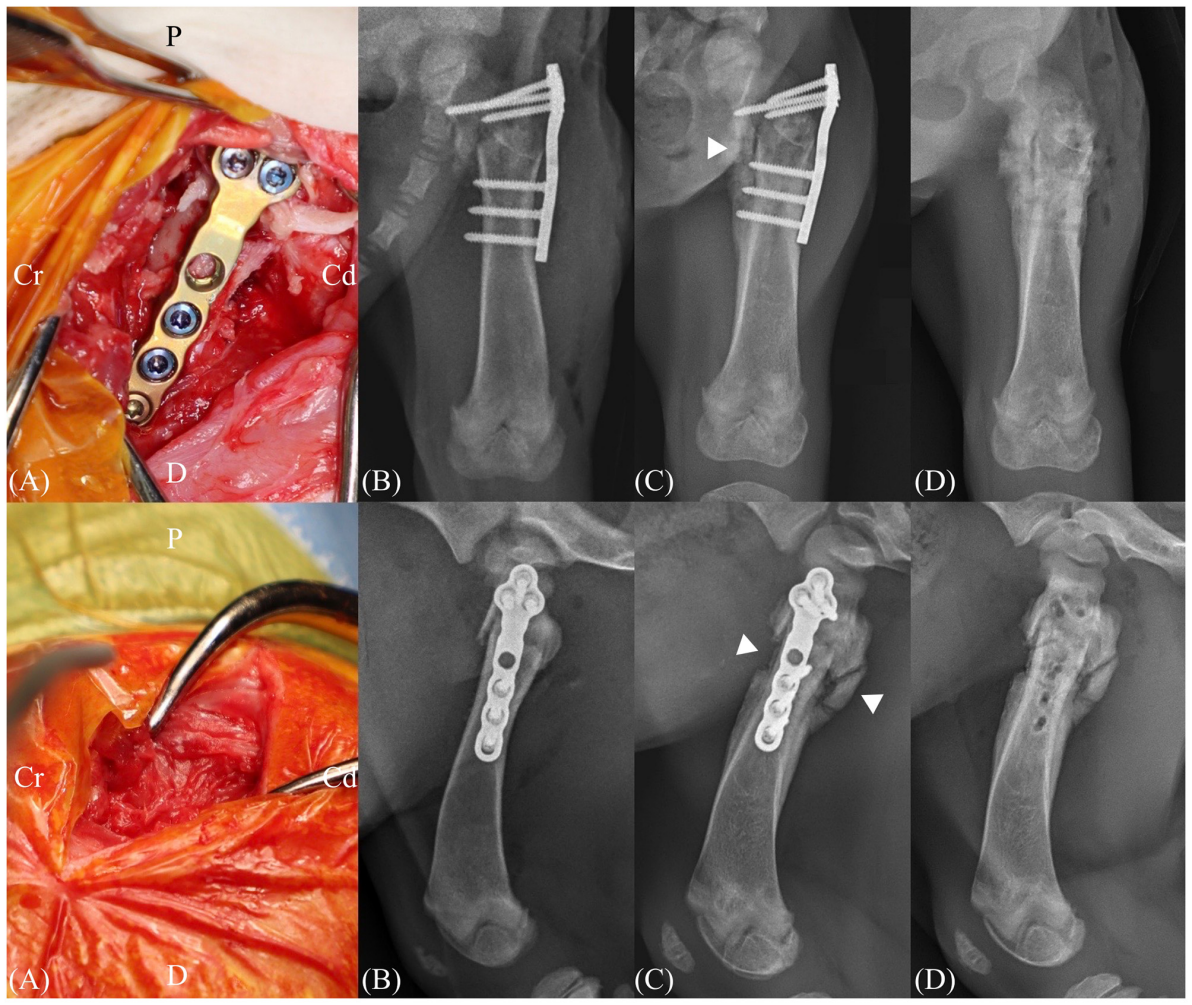
Intraoperative images and postoperative radiographs. **(A)** A tibial plateau leveling osteotomy (TPLO) plate was placed over the supratrochanteric region and secured using locking screws. **(B)** Immediate postoperative radiography images after subtrochanteric fracture repair. **(C)** Radiographs obtained 16 days postoperatively demonstrate bridging callus formation on three out of four cortices (arrowhead). **(D)** Immediate postoperative radiography images after implant removal. Cr, cranial; Cd, caudal; D, distal; P, proximal.

The patient was positioned in right lateral recumbency, and a craniolateral approach to the hip and proximal femur was performed to expose the surgical field. Considering the pediatric nature of the patient, soft tissue dissection was minimized, with particular attention paid to preserving tendon attachments in the trochanteric region. Hematoma at the fracture site was also preserved as much as possible. Fracture reduction was achieved by gently manipulating the bone using bone holding forceps, as excessive pressure could have resulted in iatrogenic fracture due to the fragility of the immature bone. The reduction was performed to restore overall femoral alignment, rather than achieving complete anatomic reconstruction. A 2.0 TPLO plate (Jeil Medical, Seoul, Republic of Korea) was contoured to match the anatomical shape of the trochanteric and subtrochanteric region. The plate was applied on the lateral side of the proximal femur and fixed in a neutralization fashion using 3 locking screws in the proximal segment and two locking screws and one cortical screw in the distal segment (Figure 2). Soft tissues were routinely closed in layers, including the subcutaneous tissue and skin. The total duration of the surgery was approximately 50 min.

Remifentanil (6–18 μg/kg/h IV) was administered for postoperative analgesia for 24 h, and Cefazolin (22 mg/kg IV, q12h) was administered as postoperative antibiotics for 3 days. Bacterial culture and antibiotic susceptibility testing of the surgical site yielded negative results.

Postoperative radiographs were obtained immediately after surgery confirmed acceptable overall femoral alignment, although complete anatomic reconstruction of the femur was not achieved.

The patient began to bear weight on the left pelvic limb 3 days after surgery. Follow-up radiographic examinations were performed on postoperative days 5, 11, and 16 to evaluate bone healing. Radiographic evidence of callus formation was first observed on postoperative day 5 and progressively increased to achieve bridging callus formation at 3 out of 4 cortices on anteroposterior and lateral views on postoperative day 16 (Figure 2). Implant removal was performed 16 days following initial surgery. Callus formation was observed around the fracture site intraoperatively, and both the screws and plate were removed. No gait abnormalities were observed following implant removal, and the patient was discharged at 7 days following bone plate removal.

**Figure 3:**
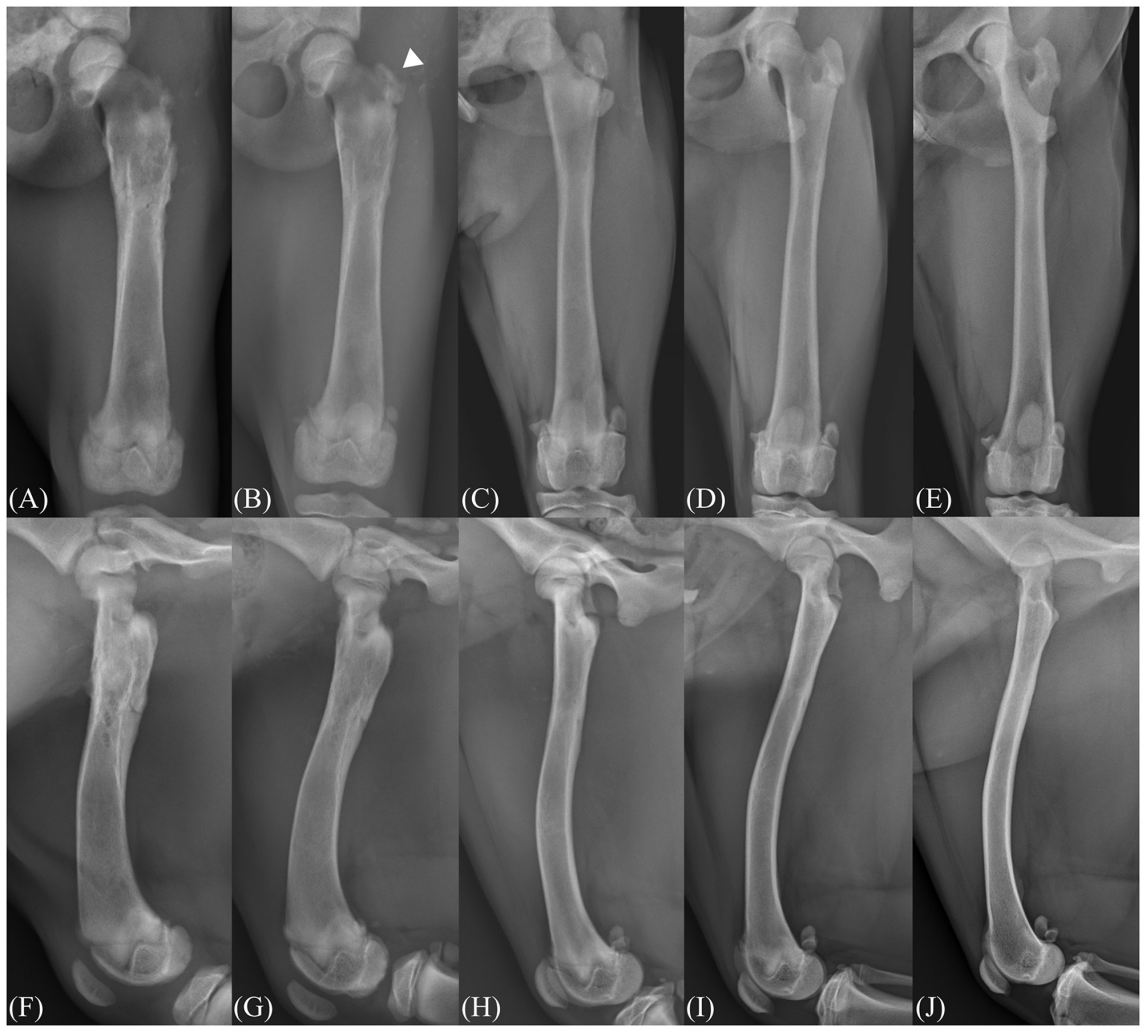
Radiographic follow-up images. Postoperative follow-up radiography images taken on 1 month **(A, F)**, 2 months **(B, G)**, 3 months **(C, H)**, 6 months **(D, I)**, 15 months **(E, J)** postoperatively. The development of the greater trochanter and the trochanteric apophysis is visible at 2 months postoperatively (arrowhead).

Follow-up evaluations were performed at 1, 2, 3, 6, 9, and 15 months postoperatively. Radiographic evidence of initial greater trochanter development was first noted 2 months following surgery (Figure 3). At the time of the final follow-up conducted 15 months postoperatively, complete closure of the proximal femoral physis was confirmed, indicating skeletal maturity. At that time, the radiographic morphology of the greater trochanter was comparable to that of the contralateral limb in both shape and size. Bilateral femoral lengths, measured in accordance with established methods, showed 186.1 mm on the right and 177.7 mm on the left, indicating no clinically relevant discrepancy (10).

In addition to femoral length, morphometric evaluation of femoral geometry was conducted using previously described radiographic landmarks (7), including the articulotrochanteric distance (ATD), and inclination angle. At the final follow-up, the ATD was measured as 35.7 mm on the right and 33.6 mm on the left, while the inclination angle was 128.7° on the right and 130.8° on the left. These findings demonstrated minimal side-to-side differences, indication no clinically relevant femoral deformities associated with the surgical intervention.

**Figure 4:**
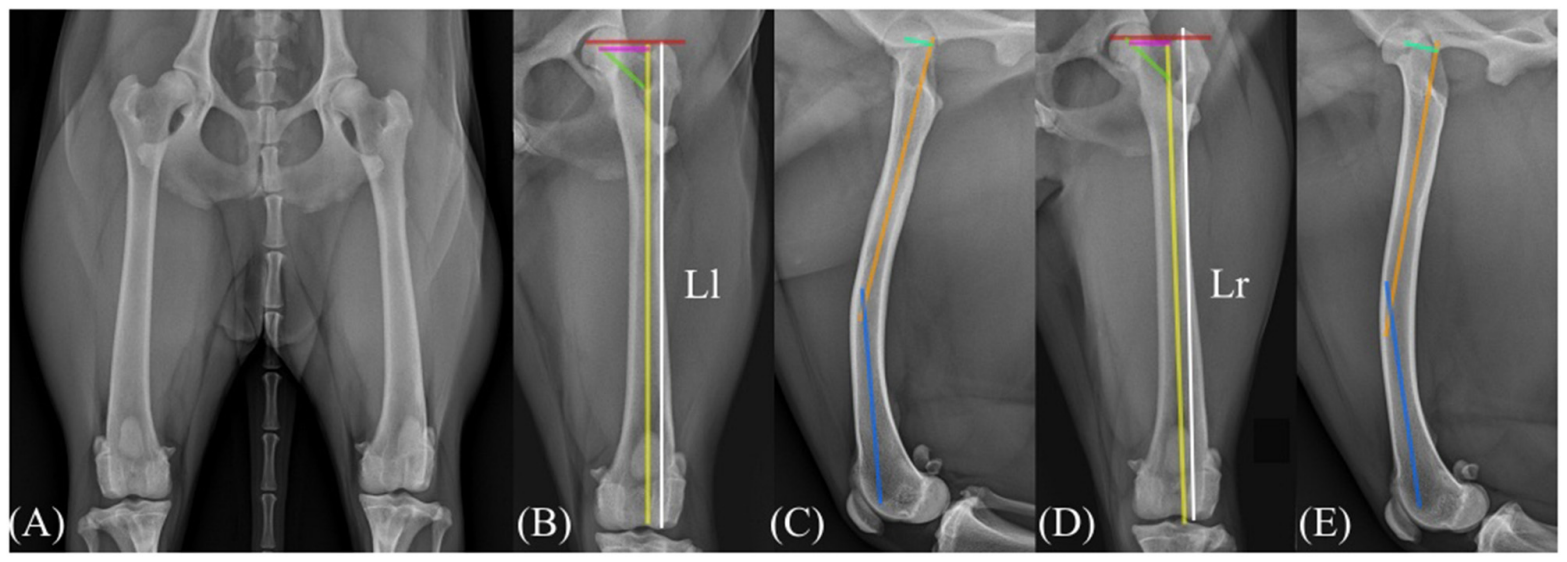
Radiographic images 15 months following surgery. Anteroposterior radiographs of both pelvic limbs demonstrate comparable shape and size of the greater trochanters on the right and left femurs **(A)**. Radiographic evaluation of the left femur **(B, C)** and right femur **(D, E)** showed femoral lengths of 186.1 and 177.7 mm, respectively (Ll, Length of the left femur; Lr, length of the left femur). In panels **(B)** and **(D)**, the red line indicates the articulotrochanteric distance (ATD), the yellow line represents the anatomical axis of the femur, and the green line bisects the femoral neck. The femoral inclination angle was calculated as the angle between the yellow and green lines. The ATD and inclination angle were 33.6 mm and 130.8° in the left femur, and 35.7 mm and 128.7° in the right femur, respectively. In panels **(C)** and **(E)**, the orange line represents the proximal anatomic axis (PAA), and the blue line represents the distal anatomic axis (DAA) used to determine the femoral procurvatum angle, which measured 157.7° in the left femur and 159.9° in the right femur. The light-green lines indicate the *x*-ais distance, and the pink lines in panels **(B)** and **(D)** indicate the *y*-axis distance used for calculating the femoral anteversion angle according to the biplanar method. The calculated anteversion angles were 22.2° for the left femur and 30.6° for the right femur.

The femoral procurvatum angle, measured as the angle between the proximal and distal anatomic axes (PAA-DAA) (11), was 160° on the right and 157.7° on the left, indicating a negligible side to side difference. The femoral anteversion angle was calculated using the biplanar trigonometric (θ = arctan [*x*/*y*]) (11), based on the distance from the center of the femoral head to the anatomic axis in the sagittal (*x*) and frontal (*y*) planes. The calculated anteversion angles were 30.6° for the right femur (*x* = 10.9 mm, *y* = 18.4 mm) and 22.2° for the left femur (*x* = 8.4 mm, *y* = 20.6 mm), demonstrating a mild decrease in anteversion on the affected side. Thigh girth measurements were comparable between the left and right pelvic limbs (Figure 4). The range of motion of the hip joint was 43°-162°, and that of the stifle joint was 35°-153° in the affected limb (Table 1).

**Table 1:** Morphometric comparison of affected and contralateral femurs at 15 months postoperatively.

**Parameter**	**Right (contralateral)**	**Left (affected)**	**Difference**
Femoral length (mm)	186.1	177.7	−8.4
Articulotrochanteric distance (mm)	35.7	33.6	−2.1
Inclination angle (°)	128.7	130.8	+2.1
Procurvatum angle (°)	159.9	157.7	−2.2
Anteversion angle (°)	30.6	22.2	−8.4

All radiographic measurements were performed independently by three observers using digital radiographic software. Each parameter was measured three times, and the mean of the three measurements was used for analysis to minimize interobserver variability.

Throughout the follow-up period, the patient exhibited a normal gait pattern, and pressure plate analysis (FMD system, Zebris Medical GmbH, Isny im Allgau, Germany) revealed peak vertical force during the stance phase of walking of 54 percent body weight (% BW) in the right pelvic limb, and 49% BW in the left pelvic limb, demonstrating nearly symmetrical weight bearing (Supplementary Video S1).

## 3 Discussion

Temporary supratrochanteric plate fixation was applied for the surgical management of a subtrochanteric femoral fracture in an immature dog, where secure fixation was complicated by the limited bone stock proximal to the fracture and the absence of a developed trochanteric apophysis. Despite the challenges associated with skeletal immaturity and the anatomical constraints of the proximal femur, satisfactory fracture stabilization and bone healing were achieved. Long-term follow-up revealed symmetric limb function and development, with no evidence of clinically relevant femoral deformities or gait abnormalities.

Various fixation techniques have been reported for the treatment of subtrochanteric femoral fracture, including interlocking nail, plate fixation, and external skeletal fixation (2, 3). The selection of an appropriate technique depends on multiple factors, such as the patient's age, fracture configuration, and the anatomical characteristic of the proximal segment. Interlocking nailing and plate fixation are commonly employed techniques for managing subtrochanteric femoral fractures, with interlocking nails being particularly favored due to their superior biomechanical properties (12). In this case involving an immature dog, interlocking nailing and external skeletal fixation requiring a tie-in configuration were considered inappropriate due to the risk of femoral head avascular necrosis and hip joint alteration, which is associated with intramedullary pinning in skeletally immature patients (13, 14).

In plate osteosynthesis for immature patients, transphyseal screw placement is generally avoided due to the risk of physeal arrest, which may lead to limb length discrepancy and angular limb deformity (5, 15). From this perspective, cranial plating avoids transphyseal screw placement, but it typically requires extensive dissection of the vastus lateralis muscle. In particular, surgical manipulation near the proximal femoral physis may compromise the ascending branch of the medial femoral circumflex artery or epiphyseal vessels, potentially leading to avascular necrosis of the femoral head.

In contrast, lateral plating carries the potential of damaging the trochanteric growth plate if screws are placed across the trochanteric apophysis. To mitigate this risk, a previous report of immature subtrochanteric femoral fracture employed a double hook plate on the lateral side of the femur to avoid injury to the trochanteric physis (2). However, in the present case, a double-hook plate was not available, and the absence of a clearly formed trochanteric apophysis made it difficult to identify and avoid the prospective physeal region during plate placement. In addition, the fracture configuration left a small proximal segment with limited bone stock, which further restricted the possibility of selective screw placement. Therefore, an alternative approach using temporary supratrochanteric plating was chosen to achieve stable fixation without permanent physeal damage.

Temporary transphyseal screw fixation has been reported to induce only reversible physeal injury, with growth resuming following implant removal (16). In contrast, the greater trochanteric apophysis develops through tensile forces generated by the muscles inserting on the greater trochanter. In human medicine, these forces change with age and muscular development (17). Although comparable biomechanical data is not available in dogs, similar age-related alterations in muscular traction are likely to occur. Considering this and the patient's young age and high regenerative potential, a looking plate was temporarily applied to the proximolateral aspect of the femur, with screws placed across the trochanteric region. Given the patient's young age and high regenerative potential, rapid bone healing was anticipated, and it was expected that apophyseal and greater trochanter development would proceed normally due to the tensile forces generated by the intact gluteal muscle attachments following implant removal.

Plate fixation of subtrochanteric femoral fractures presents inherent challenges due to the small size of the proximal fragment and the anatomical curvature of the proximal femur (4). In human pediatric patients, various anatomical plates, such as the proximal femoral locking compression plate and the proximal humerus locking plate, as an alternative, have been employed to address these difficulties (18, 19). Similarly, in the present case, a tibial plateau leveling osteotomy (TPLO) plate was utilized for the repair of a pediatric subtrochanteric femoral fracture. This approach allowed for the placement of three locking screws within the limited proximal segment, providing adequate mechanical stability while achieving sufficient bone-to-plate contact with minimal contouring. The TPLO plate may serve as a viable alternative to conventional straight or T-shaped plates for fixation of pediatric subtrochanteric femoral fractures.

Once bridging callus formation was observed on three out of four cortices on orthogonal radiographic views, the plate and screws were removed, and the bone healing continued after implant removal. On long-term follow-up through skeletal maturity, the patient showed no clinically significant limb length discrepancy or angular limb deformity. In addition, radiographic evaluation confirmed normal formation of the greater trochanter on the affected limb, with no evidence of abnormal hip joint conformation. These findings suggest that temporary supratrochanteric plating performed prior to apophyseal development may allow immature subtrochanteric femoral fractures to heal without compromising physeal growth potential.

In the present case, a mild procurvatum deformity was identified on the follow-up radiographs and was presumed to have resulted from cranial cortical malposition observed in the immediate postoperative images. This malposition likely occurred due to limited surgical manipulation and may have contributed to the progressive anterior curvature of the femur as growth continued postoperatively. At the final evaluation, the procurvatum angle measured 21.8°, representing a 7.9% increase compared to the contralateral limb. No lameness or limited range of motion was observed, suggesting that the degree of procurvatum was clinically insignificant in this patient.

This case report has several limitations. First, the effects of injury to the greater trochanteric region prior to apophyseal development on growth potential and its underlying mechanisms have not been thoroughly investigated. In particular, the histological and biomechanical responses of the developing apophyseal cartilage to temporary plate application remain unclear and warrant further investigation. Second, the timing of apophyseal development is difficult to determine, as skeletal maturity varies among individuals and can be influenced by external factors such as nutrition and mechanical loading (20). Third, although the implant was removed after confirmation of bridging callus formation, the risk of refracture remains a limitation, given that the mechanical strength of the bone is not fully restored at the stage of hard callus formation. Lastly, this report describes a single case, and further studies with larger case numbers are required to validate the applicability of the technique.

## 4 Conclusion

This case report describes the successful management of a subtrochanteric femoral fracture in an immature dog using temporary supratrochanteric plating prior to the development of the trochanteric apophysis. Radiographic and clinical follow-up confirmed normal skeletal development, including symmetrical femoral growth and appropriate formation of the greater trochanter. These findings suggest that, when carefully timed and executed, temporary fixation across the trochanteric region may serve a feasible option for stabilizing proximal femoral fractures in skeletally immature dogs with insufficient proximal bone stock, offering rigid fixation while minimizing the risk of permanent physeal disturbance.

## Patient perspective

The owner reported that the patient returned to normal activity levels and showed no signs of discomfort following recovery. The cosmetic and functional outcomes were satisfactory, and the owner expressed high satisfaction with the surgical outcome.

## Data Availability

The original contributions presented in the study are included in the article/Supplementary material, further inquiries can be directed to the corresponding author.

## Supplementary material

Supplementary Video S1. Postoperative gait analysis.
